# Research on Temperature Variation during Coal and Gas Outbursts: Implications for Outburst Prediction in Coal Mines

**DOI:** 10.3390/s20195526

**Published:** 2020-09-27

**Authors:** Chaolin Zhang, Enyuan Wang, Jiang Xu, Shoujian Peng

**Affiliations:** 1 Key Laboratory of Gas and Fire Control for Coal Mines, Ministry of Education, China University of Mining and Technology, Xuzhou 221116, China; weytop@cumt.edu.cn; 2 School of Safety Engineering, China University of Mining and Technology, Xuzhou 221116, China; 3 State Key Laboratory of Coal Mine Disaster Dynamics and Control, Chongqing University, Chongqing 400030, China; jiangxu@cqu.edu.cn (J.X.); sjpeng@cqu.edu.cn (S.P.)

**Keywords:** coal and gas outburst, coal temperature, concentration stress, outburst prediction, outburst prevention

## Abstract

Coal and gas outbursts are among the most severe disasters threatening the safety of coal mines around the world. They are dynamic phenomena characterized by large quantities of coal and gas ejected from working faces within a short time. Numerous researchers have conducted studies on outburst prediction, and a variety of indices have been developed to this end. However, these indices are usually empirical or based on local experience, and the accurate prediction of outbursts is not feasible due to the complicated mechanisms of outbursts. This study conducts outburst experiments using large-scale multifunctional equipment developed in the laboratory to develop a more robust outburst prediction method. In this study, the coal temperature during the outburst process was monitored using temperature sensors. The results show that the coal temperature decreased rapidly as the outburst progressed. Meanwhile, the coal temperature in locations far from the outburst mouth increased. The coal broken in the stress concentration state is the main factor causing the abnormal temperature rise. The discovery of these phenomena lays a theoretical foundation and provides an experimental basis for an effective outburst prediction method. An outburst prediction method based on monitoring temperature was proposed, and has a simpler and faster operation process and is not easily disturbed by coal mining activities. What is more, the critical values of coal temperature rises or temperature gradients can be flexibly adjusted according to the actual situations of different coal mines to predict outbursts more effectively and accurately.

## 1. Introduction

Coal and gas outbursts (hereinafter referred to as outbursts) in coal mines are complex dynamic phenomena characterized by a sudden and violent ejection of large amounts of coal and gas from the working face into a limited working space within a short time [1,2,3,4]. The pulverized coal and gas flow induced by an outburst generates an enormous amount of energy [5,6,7]. This can induce severe consequences: (1) the large energy output of the coal and gas flow may directly cause underground worker casualties or equipment damage; (2) the airflow formed by the instantaneous high-pressure gas can generate turbulence in roadways; (3) high concentrations of gas may be produced within a short time, putting workers at risk of suffocation; and (4) the gas may cause further explosions if its concentration reaches the explosion limit, which can lead to secondary disasters [8,9,10,11]. Therefore, these types of outbursts remain among the most severe disasters affecting the safety of coal mines around the world.

**Figure 1:**
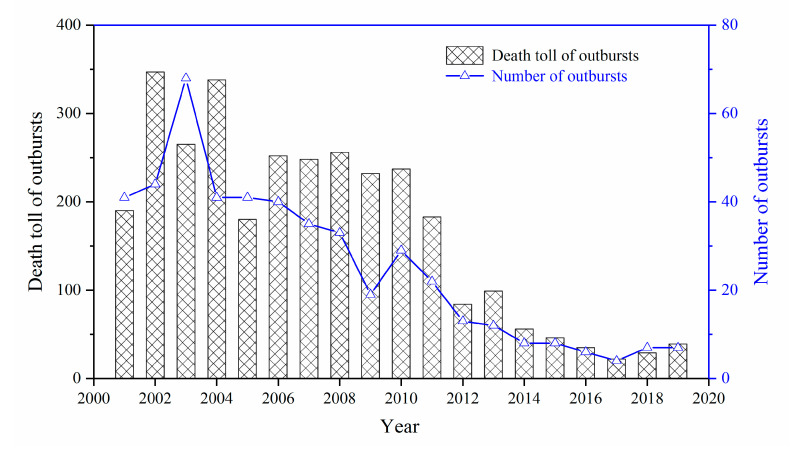
Death toll and number of outburst accidents from 2001 to 2019 in China.

In 1834, the first outburst accident was reported in France. Since then, more than 40,000 outburst accidents have been reported worldwide. Nearly half of these accidents occurred in China; therefore, China is considered the country with the highest risk of outbursts [12,13,14]. Figure 1 shows the death toll caused by outbursts and the number of outbursts in China from 2001 to 2019. The trend indicates that significant progress has been made in their prevention and control, as the number of disasters and deaths has decreased considerably since 2000. However, in recent years the depth and intensity of mining has escalated, continuously enhancing the gas content and pressure, and the geologic structures are becoming more complicated [15,16,17]. Thus, the risk of outbursts is increasing, and outburst prevention is crucial. Despite the relative progress in preventing outbursts in China over the past 20 years, the death toll of the outbursts has increased continuously since 2017. A total of seven outburst accidents occurred in 2019, resulting in 39 deaths, which is almost double that from 2017. Therefore, outbursts remain among the most pressing problems in coal mine safety in China. Hence, it is necessary to investigate a more effective method for outburst prediction.

Numerous researchers have conducted studies on outburst prediction. Outburst prediction methods are mainly divided into contact and non-contact methods [18]. Contact prediction methods include the drill cuttings gas index method (DCG), the drilling cuttings weight index method (DCW), the gas pressure method (*P*), the hardiness coefficient method (*f*), the *R*-value index method, and the composite index method (*D* and *K*) [19,20,21,22]. However, these methods have heavy workloads and long prediction times, which negatively impacts coal production. Further, they are empirical or based mainly on localized data. For example, using the gas pressure method, the coal seam is considered to have an outburst potential as long as *P* ≥ 0.74 MPa; however, this omits the fact that the geological conditions of coal mines vary among different areas. In fact, several outburst accidents have occurred at a low gas pressure, indicating the incomplete accuracy of the above prediction indicators. With the development of geophysical monitoring technology, an increasing number of non-contact methods for outburst prediction have been proposed, such as the ultrasonic testing method, the electromagnetic radiation (EMR) method, the acoustic emission (AE) method, the desorption rate index (DRI), and the micro-shock method [23,24,25,26,27]. However, even though non-contact technologies have been commercialized, the accuracy and stability of their outburst predictions remain poor. This is mainly because the precursory characteristics of outburst signals are not entirely clear, and the signals are easily interfered with by coal mining activities [28].

The outburst process is characterized by the release of a large amount of energy, which leads to the conversion and transfer of heat, inducing a change in the coal temperature. It is difficult and unsafe to study this process directly at a coal mine site because of the sudden and hidden threat of an outburst. Therefore, we conducted the outburst tests in the laboratory, during which the spatio-temporal variation in the coal temperature was investigated. We developed a more robust outburst prediction method based on experimental results and a theoretical analysis and discussed its applications and advantages for outburst prediction in coal mines.

## 2. Experimental Method

### 2.1. Test Equipment

**Figure 2:**
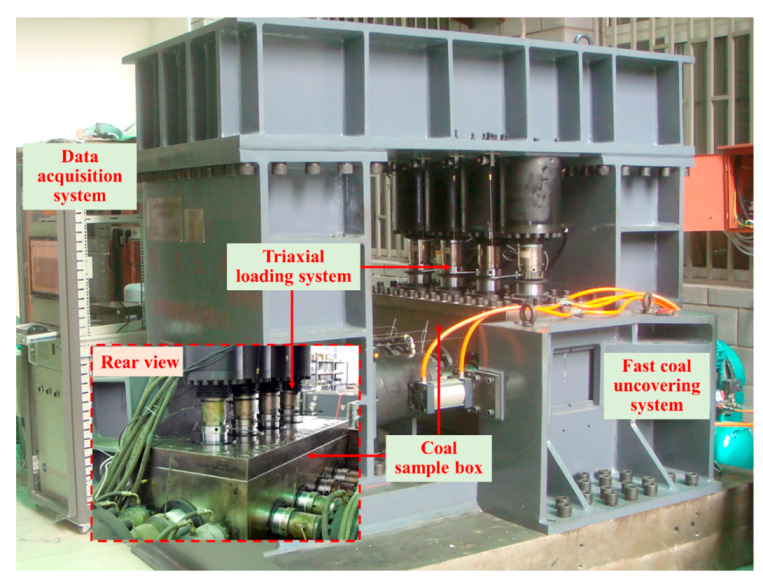
Photograph of the self-developed large-scale multifunctional equipment.

The outburst test was conducted using self-developed large-scale multifunctional equipment [29]. The LSMF equipment comprises a coal sample box, a triaxial loading system, a fast coal uncovering system, and a data acquisition system, as shown in Figure 2. The dimensions of the coal sample box were 1050 × 410 × 410 mm^3^. There was a ventilation plate on the bottom of coal sample box and there were lots of air holes distributed on the ventilation plate. There was a ventilation plate distributed with lots of air holes on the bottom of coal sample box to ensure the homogeneity of the adsorption equilibrium more efficiently. The triaxial loading system contained nine sets of oil cylinders distributed in three directions, which provided different stresses at different zones to simulate the stress concentration on the working face caused by underground mining activities. The fast coal uncovering system simulated outbursts triggered by the uncovering coal in rock cross-cuts. The temperature and gas pressure of the coal was recorded in real time.

### 2.2. Test Scheme

**Figure 3:**
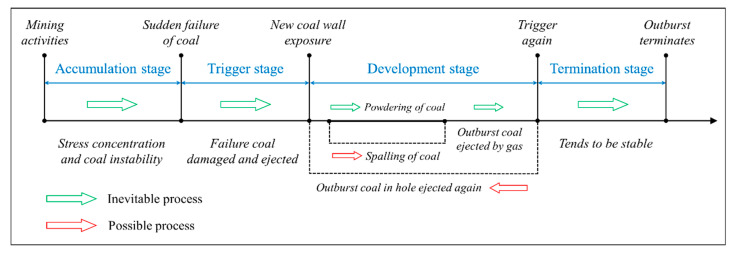
Stages of the outburst process.

**Figure 4:**
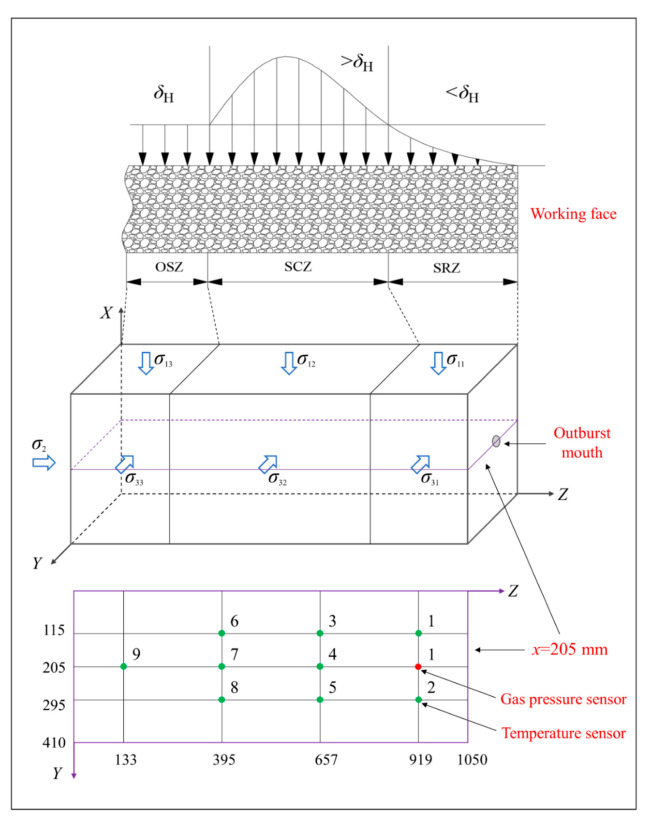
Distribution diagrams of three stress zones and temperature sensors (units: mm; *δ*_H_ means the stress in the original stress zone and *σ_ij_* means the stress loaded in three directions during experiments, *i*, *j* = 1, 2, 3).

The outburst process is typically divided into four stages—the accumulation stage, trigger stage, development stage, and termination stage [15,30,31]—as shown in Figure 3. During the outburst accumulation stage, the main indicators are the mining activities, which lead to stress concentration and coal instabilities. Three stress zones are commonly formed in front of the working face due to mining activities [32,33]—the stress relaxation zone (SRZ), stress concentration zone (SCZ), and original stress zone (OSZ)—as shown in Figure 4. The average intensity of outbursts during rock cross-cut coal uncovering is six times that of other roadways, and more than 80% of extremely severe outburst accidents occur in the process [34].

Therefore, the outburst test scheme was designed to simulate outbursts triggered by rock cross-cut coal uncovering, taking stress concentration into account. The stresses loaded in different zones in the three directions are presented in Table 1. The stress concentration factor (i.e., the ratio of stress in the SCZ to the OSZ) was 1.5, and the gas pressure was 1.0 MPa. Nine temperature sensors, labeled T1–T9, were fixed on the *x* = 205 mm plane of the coal sample to monitor the temperature variation during the outburst (Figure 4). Another temperature sensor (labeled T0) outside of the coal sample box recorded the evolution of the ambient temperature. The time constants of the temperature transmitters were 0.2s. A gas pressure sensor (labeled P1) near the outburst mouth monitored the gas pressure of the coal.

### 2.3. Test Procedure

The experimental outburst steps primarily included coal sample preparation, stress loading, vacuuming and gas injection, and the outburst trigger. A briquette coal sample was used, as large-scale raw coal samples were difficult to obtain. The raw coal sample was collected from the Sanhuiyi Coal Mine, after which it was crushed into different particle sizes. The loose coal particles with different particle mass ratios (Table 2) were pressed under 7.5 MPa using a molding machine to produce the coal specimen. The stresses at different zones were loaded synchronously after the coal sample box was sealed. The purpose of vacuuming was to ensure the purity of the adsorbed gas, and CO_2_ was used instead of CH_4_ to maximize the safety of the outburst. The CO_2_ gas reached dynamic equilibrium in the coal sample after 48 h of adsorption. Prior to triggering the outburst, the mouth was covered by two sealing plates, which were linked with two pneumatic cylinders. Once the high-pressure air in the two pneumatic cylinders was automatically discharged by the computer, the two sealing plates opened immediately, and the simulation of the outburst caused by the uncovering coal at the rock cross-cut was initialized.

## 3. Results and Discussion

### 3.1. Dynamic Evolution of the Outburst Process

**Figure 5:**
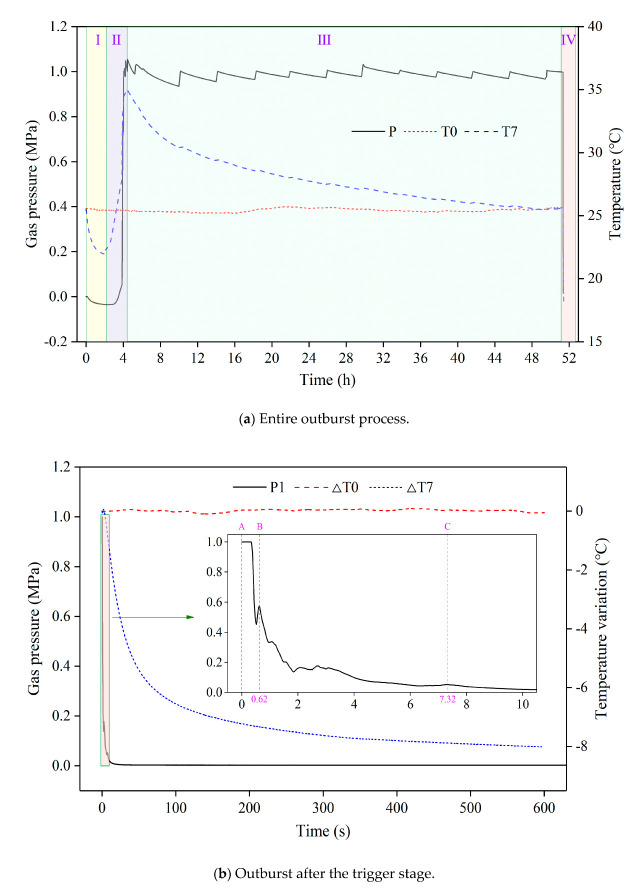
Gas pressure and temperature evolution during the outburst process.

The dynamic evolution of the gas pressure and temperature during the outburst is depicted in Figure 5a. The entire outburst process lasted nearly 52 h and can be divided into the four following sub-processes:

- Sub-process Ⅰ was the vacuuming step, lasting two hours. The gas pressure in the coal dropped from 0 to −0.035 MPa (the atmospheric pressure was defined as 0 MPa). The coal temperature decreased from 25.51 to 21.93 °C, and the ambient temperature fluctuated between 25.45 and 25.51 °C.
- Sub-process Ⅱ was the gas injection step, lasting approximately two hours. The gas pressure in the coal rebounded to 1.048 MPa. The coal temperature rebounded to 34.815 °C, and the ambient temperature fluctuated between 25.40 and 25.45 °C.
- Sub-process Ⅲ was the cyclic gas injection-adsorption step, and it lasted about 48 h [35,36]. The coal sample was circularly injected with gas in a total of 12 cycles, and each cycle lasted approximately four hours. The equilibrium pressure gradually stabilized to 1 MPa, and the coal temperature dropped to the constant ambient temperature of 25.66 °C.
- Sub-process Ⅳ was the outburst step, lasting approximately a few minutes. The gas pressure sharply dropped to 0.002 MPa. The coal temperature decreased to approximately 18 °C, and the ambient temperature remained almost constant.

**Figure 6:**
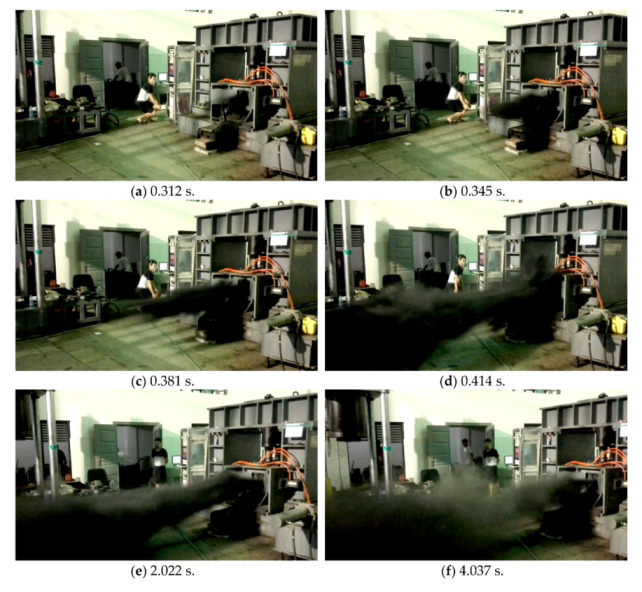

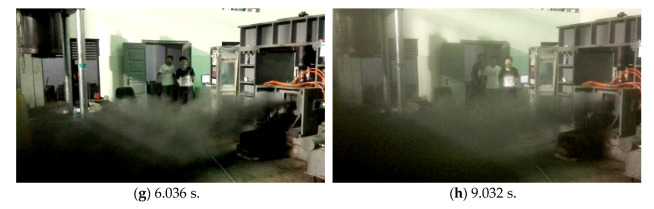
Pictures of the outburst observed at different times.

Compared with the four stages of outburst in Figure 3, sub-processes Ⅰ, Ⅱ, and Ⅲ in Figure 5a can be regarded as the accumulation stage, while sub-process Ⅳ includes the trigger stage, development stage, and termination stage. From the perspective of time, the accumulation stage is significantly longer than the three other stages. Specifically, it involves the accumulation of elastic potential energy in the coal and internal energy in the gas. When the energy reaches a critical value, the outburst is triggered within a very short time once the outburst mouth is opened. Therefore, to achieve clarity, the curves of gas pressure and temperature during the trigger stage, development stage, and termination stage are enlarged in Figure 5b (the time at which the outburst mouth opened is the reference point). Photographs of the outburst observed in the lab are shown in Figure 6.

Figure 5b shows that the gas pressure near the outburst mouth dropped faster than the coal temperature, and the gas pressure curve exhibits multiple local peaks. For example, P1 falls to 0.452 MPa at 0.52 s after the outburst trigger, after which it rises to 0.576 MPa at 0.62 s, and subsequently drops again. After the outburst mouth opened, the stress of the coal in the *Z* direction changes from a two-dimensional equilibrium state to a one-dimensional unbalanced state, leading to the sudden failure of the coal. Furthermore, as the gas pressure drops gradually, part of the pulverized coal is ejected from the outburst mouth at high speeds (50–60 m/s), as shown in Figure 6. Simultaneously, the outburst hole is filled with an increasing amount of pulverized coal until the gas pressure starts to rise. The pulverized coal continues to be ejected if the gas pressure is at a high level (another outburst was triggered at point B). After the last outburst trigger at point C, the gas pressure drops nearly to atmospheric pressure, and the outburst tends to be stable. In summary, the first 0.62 s is the trigger stage, the following 6.7 s is the development stage, and the subsequent process is the termination stage. The pulverized coal flow grows gradually during the trigger stage (Figure 6a–d), and it reaches the strongest state during the development stage (Figure 6e–g). The pulverized coal flow becomes increasingly weaker during the termination stage, and there is very little pulverized coal ejected after 10 s. However, the gas continues to flow out. Further, the temperature recorded by T7 declines continuously during all three stages.

### 3.2. Spatio-Temporal Variation in Coal Temperature

**Figure 7:**
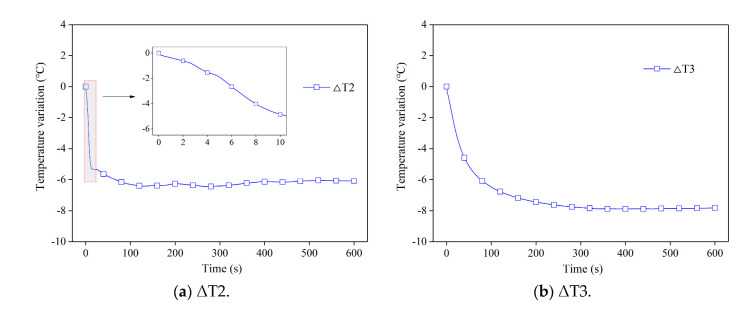

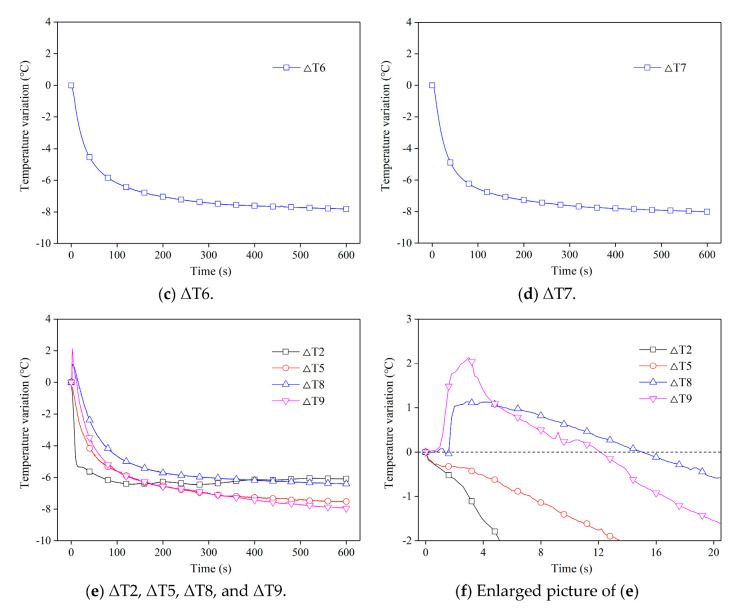
Temperature variation curves after the outburst trigger.

To analyze the temperature variations at different positions, the temperature curves of all sensors are plotted in Figure 7 (T1 and T4 were damaged during the outburst process). The figure shows that T2 drops by 5 °C during the first 10 s at a rate of about 0.5 °C/s, then drops slowly, accounting for a final net drop of 6.08 °C (Figure 7a). There are two main reasons for the temperature drop after the outburst trigger. On the one hand, gas desorption is an endothermic process, and on the other hand, the expansion of gas leads to a decrease in the coal temperature. Temperature sensor T2 was located nearest to the outburst mouth at a distance of 159 mm. Therefore, the gas adsorbed in coal at this location began to desorb initially, and the temperature near the outburst mouth had the fastest rate of decrease. Temperature sensors T3, T6, and T7 were located far from the outburst mouth, and they had a similar trend, dropping fast at first and subsequently leveling off (Figure 7b–d). However, an unusual phenomenon is observed in Figure 7e,f: the coal temperature increases for a certain period of time when the temperature sensor is further away (e.g., T8 and T9). Specifically, T8 had a maximum increment of 1.13 °C at 2.8 s, and then dropped back to 0 at 15.0 s. Similarly, T9 had a maximum increment of 2.14 °C at 3.0 s, and then dropped back to 0 at 12.2 s.

**Figure 8:**
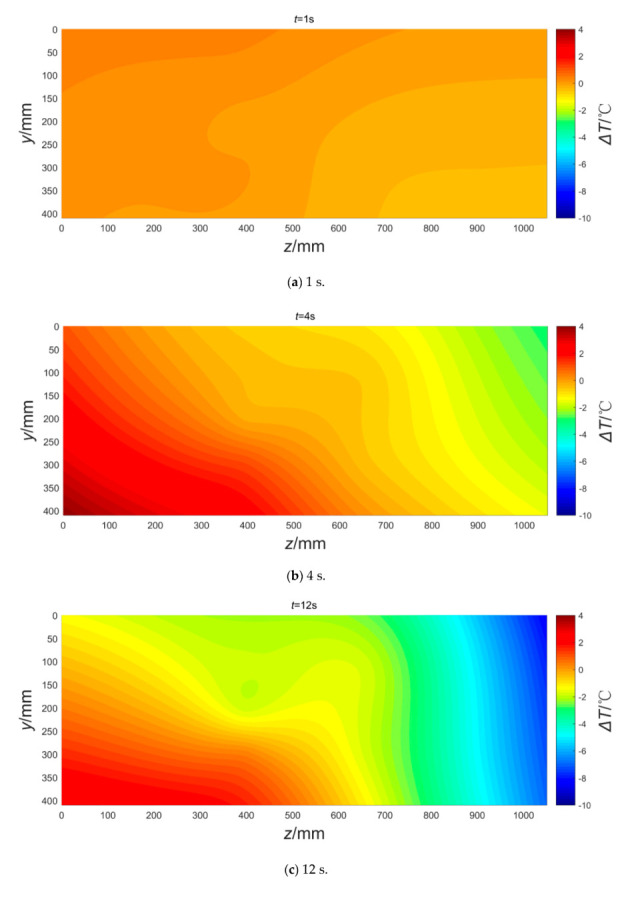

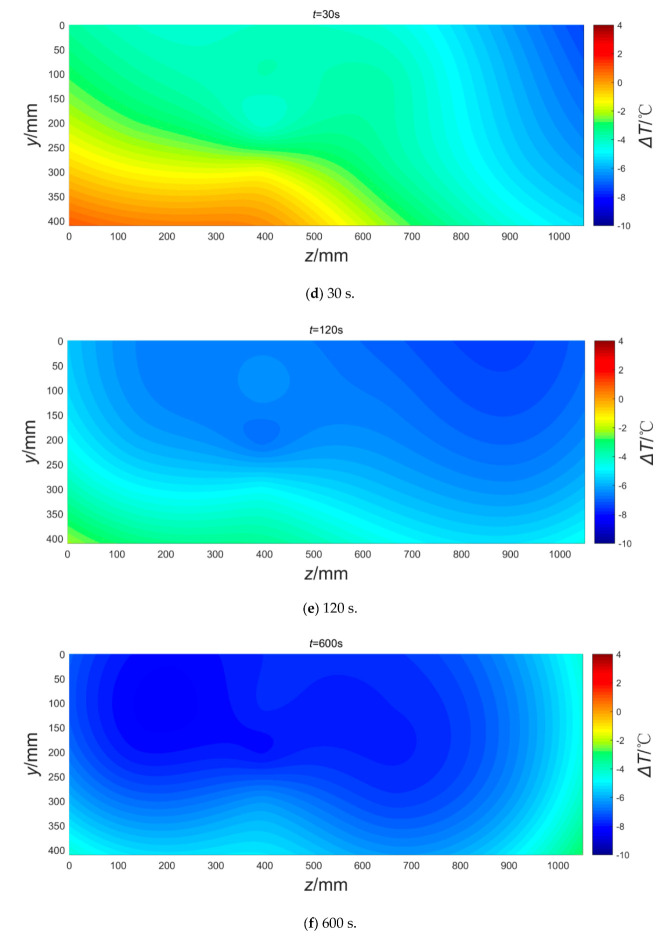
The temperature variation fields on the *x* = 205 mm plane after the outburst trigger.

The temperature variation fields on the *x* = 205 mm plane of the coal at different times were plotted using MATLAB software, as shown in Figure 8. The temperature gradient of the coal is insignificant during the first second (Figure 8a); however, the temperature of the left coal increases, whereas that of the right coal decreases after 4 s (Figure 8b). After 12 s, the rate of change of the temperature increase in the left coal decreases, whereas that of the temperature decrease in the right coal increases (Figure 8c). After 30 s, the temperature of the entire area decreases further, and the temperature drop of the right coal is significant (Figure 8d). Subsequently, the temperature gradient decreases progressively, and the temperature eventually stabilizes at 6–8 °C (Figure 8e,f). Thus, the temperature gradient at the middle of the coal is lower than that of both sides at all times, which is attributed to the smaller permeability of the SCZ zone, making it difficult for the coal to desorb gas. In summary, coal temperature evolution is a complex dynamic process, and the coal temperature drops faster when approaching the outburst. Further, the coal temperature far from the outburst mouth showed an abnormal rising.

### 3.3. Causal Analysis of Abnormal Temperature Rise

**Figure 9:**
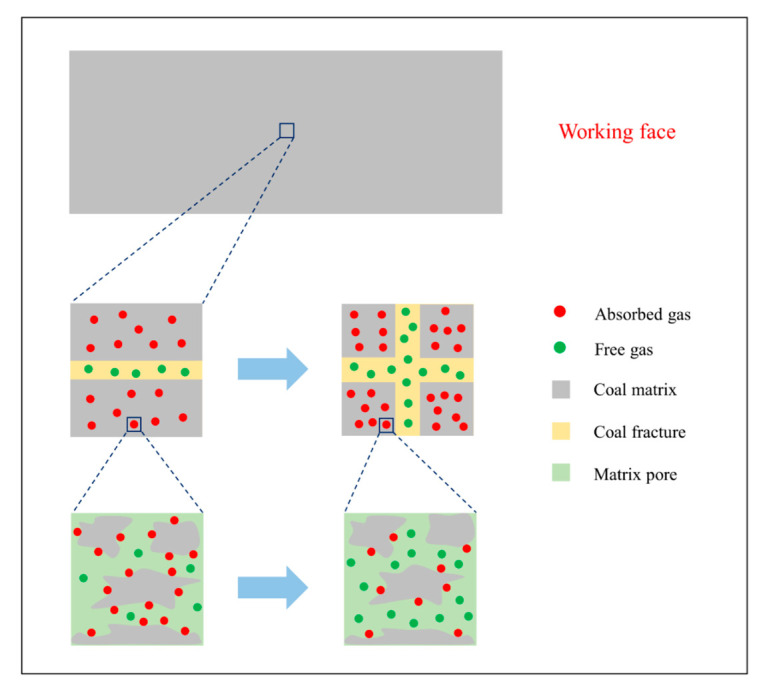
Schematic diagram of the coal thermal system.

To investigate the coal temperature rise during the outburst, a unit of coal away from the coal box boundary was considered as a thermal system, as shown in Figure 9. When coal mining activities are in progress in front of the working face, the coal is deformed and broken under the concentration stress, and the elastic potential energy stored in the coal is consumed simultaneously. Therefore, the dynamic equilibrium state of gas adsorption and desorption on the coal matrix surface is disturbed, resulting in the adsorbed gas diffusing from the matrix surface into the matrix pore and subsequently migrating into the coal fracture. The outburst is triggered immediately once the elastic potential energy of the coal and the internal energy of the free gas are larger than the critical energy, causing the coal particles and free gas to be ejected. Considering the relatively short period of outburst duration and the low thermal conductivities of coal and CO_2_, the thermal system can be approximated an adiabatic system, and the heat exchange between the thermal system and the external environment is very limited. Therefore, the factors of coal temperature variation arise from three quantities: concentrated stress work, adsorbed and free gas, and coal oxidation.

During the process of coal breaking and deformation, the work originates from stress work and the elastic potential energy stored in the coal. First, coal fractures develop and expand, resulting in an increase in the temperature at the crack tip. Second, the friction between the coal particles generates heat. Eventually, the increased coal surface also consumes part of the work. Therefore, the temperature variation can be expressed as:

(1) $$\Delta T_{1}=\frac{\Delta Q_{1}+\Delta Q_{2}}{m_{0}c_{0}}$$

where Δ*T*_1_ is the temperature variation affected by stress, °C; Δ*Q*_1_ is the heat generated at the crack-tip, J; Δ*Q*_2_ is the heat generated at the coal particle surface, J; *m*_0_ is the coal mass, kg; *c*_0_ is the specific heat capacity, J/(kg·°C).

The absorbed gas desorbing from the coal matrix and free gas expanding into the fracture are endothermic processes, which lead to the coal temperature change:

(2) $$\Delta T_{2}=-\frac{\Delta Q_{3}+\Delta Q_{4}}{m_{0}c_{0}}$$

where Δ*T*_2_ is the temperature variation affected by the gas, °C; Δ*Q*_3_ is the heat exchange due to gas desorption, J; Δ*Q*_2_ is the heat exchange due to gas expansion, J.

Coal oxidation is an exothermic chemical reaction, and therefore:

(3) $$\Delta T_{3}=\frac{\Delta Q_{5}}{m_{0}c_{0}}$$

where Δ*T*_3_ is the temperature variation affected by coal oxidation, °C; Δ*Q*_5_ is the heat generated from coal oxidation, J.

It is very difficult for oxygen in the air to flow into the outburst coal seam, as outbursts usually occur in mines at a high gas pressure. Furthermore, the coal oxidation process is an extremely slow process. Therefore, the heat and temperature affected by coal oxidation can be ignored. Consequently, the total temperature variation in the coal is mainly affected by two factors—namely, stress and gas—and thus can be expressed as:

(4) $$\Delta T=\Delta T_{1}+\Delta T_{2}$$

where ΔT is the total temperature variation in coal, °C (ΔT1 > 0 °C and ΔT2 < 0 °C).

Therefore, the coal broken in the stress concentration state is the main cause behind the abnormal temperature rise.

### 3.4. Implications for Outburst Prediction in Coal Mines

**Figure 10:**
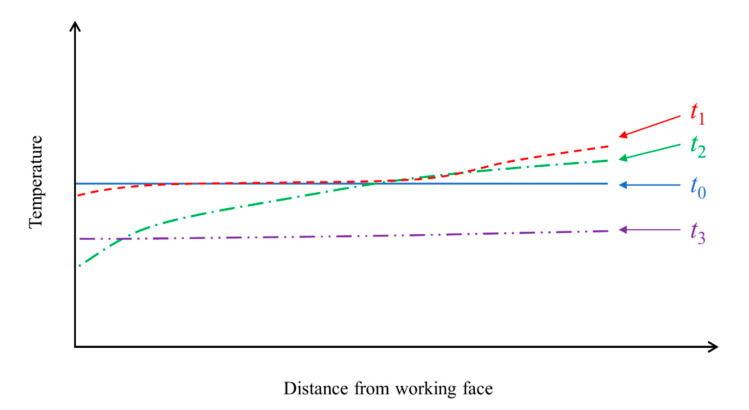
Dynamic evolution of the coal temperature at different locations.

According to the above analysis, the processes of coal breaking, gas desorption, and gas expansion are synchronous, and they exert different thermal effects. Therefore, the macroscopic coal temperature change mainly depends on the dominant factor. The following four stages are summarized based on the experimental results and theoretical analysis, as shown in Figure 10.

- Stage *t*_0_. The stress and gas are both in equilibrium before the coal mining activities begin. The coal temperatures at different locations are similar, and there is no obvious temperature gradient.
- Stage *t*_1_. The coal mining activities deform and break coal under a large stress concentration. In the area close to the working face, the gas thermal effect is dominant (|Δ*T*_1_| < |Δ*T*_2_|), and the coal temperature drops initially. However, in the area far from the working face, the stress thermal effect is dominant (|Δ*T*_1_| > |Δ*T*_2_|), as the gas has not yet been desorbed, and the coal temperature rises initially.
- Stage *t*_2_. The outburst will be triggered if mining activities continue, and the energy reaches the critical value. Then, an increasing amount of gas is desorbed, leading to a temperature drop closer to the working face (|Δ*T*_1_| << |Δ*T*_2_|), and a large amount of coal particles and gas are ejected.
- Stage *t*_3_. After a period of outburst, the coal temperature reaches a new equilibrium state, and there is no significant temperature gradient.

Thus, the key for outburst control is preventing stage *t*_1_ from developing further into stage *t*_2_. If the abnormal rise in the coal temperature is monitored and detected in time, then the mining activities can be stopped immediately before taking outburst prevention measures, turning stage *t*_1_ back into stage *t*_0_ to prevent an outburst.

**Figure 11:**
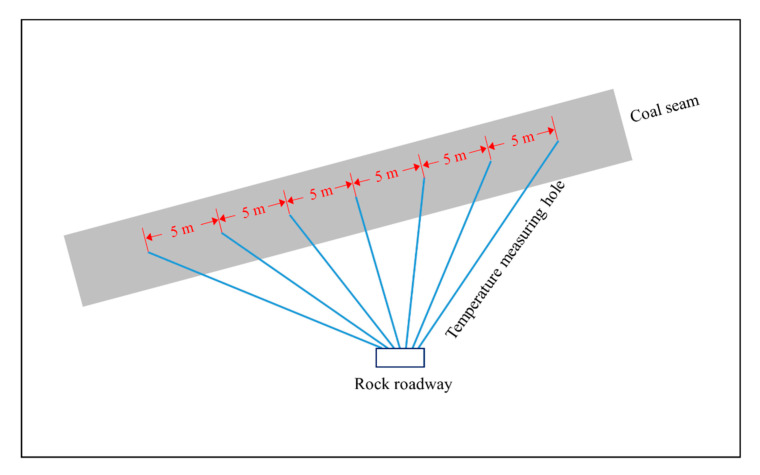
Schematic diagram of outburst prediction based on coal temperature monitoring.

Figure 11 shows the schematic diagram of outburst prediction based on coal temperature monitoring. Several holes are drilled from the rock roadway to the coal seam for temperature measurement, and the distance between the bottom two adjacent holes is 5 m. Hence, both the coal temperature, which reflects the overall temperature of the coal seam, and the coal temperature gradient, which reflects the local temperature change in the coal seam, are obtained in real time. Mining activities must be stopped immediately if the coal temperature becomes abnormal—namely, if the coal temperature rise or temperature gradient exceeds the critical values. If the danger is identified, then outburst prevention measures, such as gas drainage, protective seam mining, and water injection [37,38,39], must be applied until the threat of an outburst is eliminated.

The outburst prediction method based on the coal temperature proposed in this study has the following advantages: (1) the coal temperature measurement by drilling holes in coal seam is simple and fast; (2) the coal temperature at different positons can be monitored in real time, and it is not easily disturbed by coal mining activities; (3) the critical values of coal temperature rise or temperature gradient can be flexibly adjusted according to the actual situations of different coal mines to predict outbursts more effectively and accurately. However, its effectiveness is not confirmed in coal mines, mainly due to the uncertainty of outbursts and the long term of the monitoring cycle. Therefore, the following step must focus on how to verify the validity in the field, and combine other methods, both contact and non-contact, to achieve more accurate and efficient outburst predictions.

## 4. Conclusions

The outburst experiment was carried out to explore a novel outburst prediction method. The key findings are summarized as follows.

(1) After an outburst trigger, the coal particles and gas are ejected from the outburst mouth at high speeds (50–60 m/s) in a process lasting approximately 10 s. The coal temperature and gas pressure decrease rapidly during this process. However, the coal temperature far from the outburst mouth increases first and subsequently decreases, which is an unusual phenomenon.
(2) The factors of coal temperature variation include three quantities: concentrated stress work, adsorbed and free gas, and coal oxidation. The temperature rise is caused by the coal broken under the stress concentration state, whereas the main reason behind the temperature decrease is the absorbed gas, which is desorbed from the coal matrix, and the free gas that expands into the fracture. The effect of coal oxidation on the temperature may be ignored.
(3) The key to outburst control is preventing stage *t*_1_ from developing into stage *t*_2_. Thus, a novel outburst prediction method based on coal temperature monitoring is proposed, where both the coal temperature and its gradient are obtained in real time. Mining activities must be stopped immediately if the coal temperature rise or temperature gradient exceed the critical values.
(4) The outburst prediction method based on the coal temperature proposed in this study has many advantages compared to the traditional prediction methods. Future studies will investigate how to verify its validity in the field, and combine other methods, both contact and non-contact, to achieve more accurate and efficient outburst predictions.

## Figures and Tables

**Table 1:** Stress loading of the outburst at different zones.

Stress Zones	OSZ	SCZ	SRZ
*σ*_1_/MPa	2.0	3.0	1.0
*σ*_2_/MPa	2.0	2.0	2.0
*σ*_3_/MPa	1.2	1.8	0.6

**Table 2:** Particle size of the briquette coal sample.

Particle Size/mm	0–0.15	0.15–0.18	0.18–0.25	0.25–0.425	0.425–0.85	0.55–2.0
Particle mass ratio/%	27	3	5	11	19	35

## References

[1] Hudecek V. (2008). Analysis of safety precautions for coal and gas outburst-hazardous strata. J. Min. Sci..

[2] Jin K., Cheng Y., Ren T., Zhao W., Tu Q., Dong J., Wang Z., Hu B. (2018). Experimental investigation on the formation and transport mechanism of outburst coal-gas flow: Implications for the role of gas desorption in the development stage of outburst. Int. J. Coal Geol..

[3] Dennis J. (2019). Review of coal and gas outburst in Australian underground coal mines. Int. J. Min. Sci. Technol..

[4] Goran V., Maja K., Milivoj V. (2016). Study of coal burst source locations in the Velenje colliery. Energies.

[5] Busch A., Gensterblum Y., Krooss B. (2003). Methane and CO_2_ sorption and desorption measurements on dry Argonne premium coals: Pure components and mixtures. Int. J. Coal Geol..

[6] Wold M., Connell L., Choi S. (2008). The role of spatial variability in coal seam parameters on gas outburst behavior during coal mining. Int. J. Coal Geol..

[7] Lu S., Wang C., Liu Q., Zhang Y., Liu J., Sa Z., Wang L. (2019). Numerical assessment of the energy instability of gas outburst of deformed and normal coal combinations during mining. Process Saf. Environ. Prot..

[8] Lama R., Bodziony J. (1998). Management of outburst in underground coal mines. Int. J. Coal Geol..

[9] Sobczyk J. (2014). A comparison of the influence of adsorbed gases on gas stresses leading to coal and gas outburst. Fuel.

[10] Nilufer K., Mustafa O. (2019). Application of structural equation modeling to evaluate coal and gas outbursts. Tunn. Undergr. Space Technol..

[11] Fu G., Xie X., Jia Q., Tong W., Ge Y. (2020). Accidents analysis and prevention of coal and gas outburst: Understanding human errors in accidents. Process Saf. Environ. Prot..

[12] Fan C., Li S., Luo M., Du W., Yang Z. (2017). Coal and gas outburst dynamic system. Int. J. Min. Sci. Technol..

[13] Ye Q., Wang G., Jia Z., Zheng C., Wang W. (2018). Similarity simulation of mining crack-evolution characteristics of overburden strata in deep coal mining with large dip. J. Pet. Sci. Eng..

[14] Dmytro R., Valeriy S. (2019). A mathematical model of gas flow during coal outburst initiation. Int. J. Min. Sci. Technol..

[15] Chao J., Dai L., Sun T., Wang B., Zhao B., Yang X., Zhao X., Guo P. (2019). Experimental study of the impact of gas adsorption on coal and outburst dynamics effects. Process Saf. Environ. Prot..

[16] Zhou B., Xu J., Peng S., Yan F., Yang W., Cheng L., Ni G. (2020). Experimental Analysis of the Dynamic Effects of Coal–Gas Outburst and a Protean Contraction and Expansion Flow Model. Nat. Resour. Res..

[17] Choi S., Wold M., Stephanson O. (2004). A coupled geomechanical-reservoir model for the modelling of coal and gas outbursts. Coupled Thermo-Hydro-Mechanical-Chemical Processes in Geo-Systems: Fundamentals, Modelling, Experiments and Applications.

[18] Xie X., Fu G., Xue Y., Zhao Z., Chen P., Lu B., Song J. (2019). Risk prediction and factors risk analysis based on IFOA-GRNN and apriori algorithms: Aapplication of artificial intelligence in accident prevention. Process Saf. Environ. Prot..

[19] Gui X., Xu Y., Meng X., Yu Z. (2009). Application of the value of drilling cuttings weight and desorption index for drill cuttings to preventing coal and gas outburst. J. Univ. Sci. Technol. Beijing.

[20] Li D., Cheng Y., Wang L., Wang H., Wang L., Zhou H. (2011). Prediction method for risks of coal and gas outbursts based on spatial chaos theory using gas desorption index of drill cuttings. Min. Sci. Technol..

[21] State Administration of Work Safety (2019). Specification of Coal and Gas Outburst Prevention in China.

[22] Wang C., Li X., Xu C., Niu Y., Cheng Y., Yang S., Zhou B., Jiang C. (2020). Study on factors influencing and the critical value of the drilling cuttings weight: An index for outburst risk prediction. Process Saf. Environ. Prot..

[23] Marta K. (1997). Ultrasonic studies of outburst-prone coals. Int. J. Rock Mech. Min..

[24] Lu C., Dou L., Liu H., Liu H., Liu B., Du B. (2012). Case study on microseismic effect of coal and gas outburst process. Int. J. Rock Mech. Min. Sci..

[25] Qiu L., Song D., Li Z., Li B., Liu J. (2019). Research on AE and EMR response law of the driving face passing through the fault. Saf. Sci..

[26] Dennis J. (2017). Investigations into the identification and control of outburst risk in Australian underground coal mines. Int. J. Min. Sci. Technol..

[27] Alexandr S., Artyom K. (2020). Spectral-acoustic method for outburst danger prediction considering in-situ gas pressure. Vth Int. Innov. Min. Symp..

[28] Qiu L., Li Z., Wang E., Liu Z., Ou J., Li X., Ali M., Zhang Y., Xia S. (2018). Characteristics and precursor information of electromagnetic signals of mining-induced coal and gas outburst. J. Loss Prevent. Proc..

[29] Zhang C., Xu J., Yin G., Peng S., Li Q., Chen Y. (2019). A novel large-scale multifunctional apparatus to study the disaster dynamics and gas flow mechanism in coal mines. Rock Mech. Rock Eng..

[30] Shepeleva S., Dyrdin V. (2011). Gas emission under coal and gas outbursts. J. Min. Sci..

[31] Hu Q., Liang Y., Wang H., Zou Q., Sun H. (2017). Intelligent and integrated techniques for coalbed methane (CBM) recovery and reduction of greenhouse gas emission. Environ. Sci. Pollut. Res..

[32] Feng T., Xie X., Liu H. (2011). Experimental study of freezing temperature field in uncovering outburst coal seam in cross-cut. J. China Coal Soc..

[33] Barla G., Bonini M., Semeraro M. (2011). Analysis of the behaviour of a yield-control support system in squeezing rock. Tunn. Undergr. Space Technol..

[34] Xie H., Xie J., Gao M., Zhang R., Zhou H., Gao F., Zhang Z. (2015). Theoretical and experimental validation of mining-enhanced permeability for simultaneous exploitation of coal and gas. Environ. Earth Sci..

[35] Sobczyk J. (2011). The influence of sorption processes on gas stresses leading to the coal and gas outburst in the laboratory conditions. Fuel.

[36] Dutka B., Kudasik M., Topolnicki J. (2012). Pore pressure changes accompanying exchange sorption of CO_2_/CH_4_ in a coal briquette. Fuel Process Technol..

[37] Yin G., Li M., Jiang C., Xu J., Li W. (2015). Mechanical behavior and permeability evolution of gas infiltrated coals during protective layer mining. Int. J. Rock Mech. Min..

[38] Zhang C., Tu S., Zhang L. (2017). Pressure-relief and methane production performance of pressure relief gas extraction technology in the longwall mining. J. Geophys. Eng..

[39] Yang W., Lin M., Walton G., Lin B., Sinha S., Lu C., Li G. (2020). Blasting-enhanced water injection for coal and gas out-burst control. Process Saf. Environ. Prot..

